# A Pilot Study on the Effects of Medium-Chain Triglyceride-Enriched Oral Nutritional Supplementation on Nutritional Status, Physical Function, and Cognitive Function in Older Facility Residents

**DOI:** 10.7759/cureus.99590

**Published:** 2025-12-18

**Authors:** Mie Nakagawa, Shusaku Kanai, Astuko Kayashita, Jun Kayashita

**Affiliations:** 1 Comprehensive Scientific Research, Prefectural University of Hiroshima, Hiroshima, JPN; 2 Faculty of Health Sciences, Hiroshima Shudo University, Hiroshima, JPN

**Keywords:** bmi, long-term care health facilities, medium-chain triglycerides, muscle strength, oral nutritional supplements (ons)

## Abstract

Background

Malnutrition is associated with increased morbidity and all-cause mortality, and the proportion of undernourished individuals tends to increase with age. The Global Leadership Initiative on Malnutrition (GLIM) proposed a consensus in 2019 defining malnutrition based on criteria including weight loss, reduced muscle mass, and low body mass index (BMI). The purpose of this study was to evaluate whether daily supplementation with medium-chain triglycerides (MCTs), provided in the form of snacks, improves or maintains nutritional status in older adults who are malnourished or at risk of malnutrition, and to compare changes in muscle strength and physical function between the MCT-supplemented group and the control group. Additionally, this study aimed to assess the feasibility of incorporating MCT supplementation into routine nutritional care at long-term care facilities.

Materials and methods

The study’s participants were older residents of long-term care health facilities. The design was a pre-post comparison at a single facility. Participants were divided into a medium-chain triglycerides (MCT)-supplemented group (intervention group) and a group receiving regular meals (control group). The intervention group consumed a snack containing MCTs (135-136 kcal, 0.4 g protein) in addition to the diet provided at the facility. BMI, anthropometric measurements, the type of diet being consumed, grip strength, nutritional indicators, and cognitive function were also measured before initiation, 3 months after intake, and 5 months after intake.

Results

In the intervention group, BMI increased from 18.8 ± 2.2 kg/m² at baseline to 19.7 ± 2.3 kg/m² after 5 months (t=-3.629, p=0.002, 95% CI (−1.468 - −0.381)); BMI increased in 14/16 participants (87.5%). In the control group, BMI decreased slightly from 20.5 ± 2.8 kg/m² at baseline to 20.2 ± 2.7 kg/m² at 5 months (t=-0.767, p=0.476, 95% CI (−0.780 - 1.559)). Grip strength in the intervention group increased from 9.9 ± 3.9 kg at baseline to 11.1 ± 5.2 kg after 5 months (t=2.192, p=0.049, 95% CI (0.033 - 2.342)).MNA-SF scores improved in both the intervention group (from 7.4 ± 1.6 to 9.7 ± 1.4) and the control group (from 7.2 ± 2.1 to 9.6 ± 1.6), making it difficult to determine group-specific effects on nutritional status. No significant changes were observed in cognitive function in either group.

Conclusion

This study showed that providing one snack containing MCTs (10 g/day) daily for 5 months, in addition to regular meals, led to improvements in BMI and grip strength in undernourished older adults residing in long-term care facilities. Although MNA-SF scores also improved, the similar degree of improvement observed in both the intervention and control groups made it difficult to determine the specific contribution of MCT supplementation to overall nutritional status. Cognitive function did not show significant changes, which may be due to the short intervention period and limited sample size. Nevertheless, the observed improvements in physical function suggest that MCT-containing snacks may be a useful nutritional strategy for supporting the health of older adults. Further large-scale studies are needed to clarify the effects on cognitive function and to better distinguish the independent impact of MCT supplementation on nutritional outcomes.

## Introduction

In Japan, the 2022 edition of the White Paper on Aging reported that the proportion of older individuals in the total population had reached 28.8%, indicating a continuing upward trend [1]. Various nutrition-related issues arise as aging progresses. Many older adults who require long-term care reside in long-term care facilities and special nursing homes. While the prevalence of malnutrition among independently living older adults is less than 10%, it ranges from 27.0% to 41.0% in long-term care facilities [2]. Malnutrition is associated with increased morbidity and all-cause mortality, and the proportion of undernourished individuals tends to increase with age [3]. The Global Leadership Initiative on Malnutrition (GLIM) proposed a consensus in 2019 defining malnutrition based on criteria including weight loss, reduced muscle mass, and low body mass index (BMI) [4]. These factors are associated with sarcopenia, frailty, and increased mortality [5]. Additionally, malnutrition is associated with reduced muscle strength, physical function, and cognitive function [6]. In Japan, a study investigating factors related to the ability of residents in long-term care facilities to return home identified cognitive function and high BMI as significant predictors, highlighting the importance of improving low BMI in undernourished older individuals [7]. The provision of high-energy meals is essential [8]. Systematic reviews have demonstrated that the use of oral nutritional supplements (ONSs) for undernourished individuals in community or hospital settings can lead to reduced complications, mortality, and readmissions, and may help lower healthcare costs [9]. Oral nutritional support includes dietary counseling and meal planning assistance, food fortification, and prescribed ONSs [10]. Medium-chain triglycerides (MCTs) are considered promising nutrients for sarcopenia treatment that are known for their efficient energy utilization and rapid conversion into energy sources [11]. MCT supplementation in patients with chronic respiratory diseases and older individuals can result in weight gain and improved nutritional status [12]. However, few studies have examined whether combining nutrition counseling with medium-chain triglyceride (MCT) supplementation can improve nutritional status or physical function among older adults living in long-term care facilities. In particular, evidence remains limited regarding changes assessed using established nutritional indicators. Therefore, the objectives of this study were to evaluate whether continuous MCT supplementation provided in the form of daily snacks can improve or maintain nutritional status in older residents identified as malnourished or at risk of malnutrition, and to compare changes in muscle strength and physical function between the MCT group and the control group. Additionally, this study aimed to assess the feasibility and practicality of incorporating MCT supplementation as a routine nutritional strategy in long-term care facilities.

## Materials and methods

Participants and group allocation

Initial interest in this study was targeted toward all residents who lived in the nursing home and required special care from a helper between August 2023 and February 2024. Inclusion criteria were: (1) older adults requiring nursing care, (2) consuming ≥80% of their estimated energy requirements orally, and (3) assessed as being at risk of malnutrition. Malnutrition risk was defined as a Mini Nutritional Assessment-Short Form (MNA-SF) score of 7 or lower [13]. Participants were enrolled only after obtaining approval from their attending physicians and the facility directors, and written informed consent was obtained from all participants. Exclusion criteria included an inability to consume meals orally and unstable general health conditions. This study used a non-randomized pre-post design at a single facility. After confirming willingness to participate, residents who agreed to receive the nutritional supplement were assigned to the MCT-supplemented group (intervention group), while those who continued with routine meals comprised the control group. No randomization procedures were applied. Of the 53 residents initially eligible, 14 met the exclusion criteria. Among the remaining 39 participants, 24 were allocated to the intervention group and 15 to the control group at baseline. Participant attrition occurred in both groups. In the intervention group, eight individuals discontinued participation (five discharged home, one due to declining health, one hospitalized for a fracture, and one discharged following deterioration in condition). In the control group, six participants dropped out (two discharged home, two hospitalized for fractures, one hospitalized due to worsening illness, and one died).

Study design

A priori sample size calculation was performed using G*Power (version 3.1), based on the effect size reported in a similar study by St-Onge and Bosarge [14-15]. Assuming a two-sided t-test, an expected effect size of Cohen’s d = 0.8, a significance level of α = 0.05, and a desired power of 0.95, the required sample size was estimated to be 22 participants per group (total N = 44). Although recruitment was planned accordingly, only 39 residents met the eligibility criteria at baseline, with 24 allocated to the intervention group and 15 to the control group based on their willingness to receive MCT supplementation. During the intervention period, both groups experienced dropout due to changes in health conditions, discharge, hospitalization, or other reasons, resulting in a final analyzed sample of 16 participants in the intervention group and 9 in the control group.

Daily nutritional intake in each group

The study was conducted by dividing participants into two groups: an intervention group and a control group. All participants consumed three meals and one snack daily as routinely provided by the facilities. The intervention group additionally received a snack containing MCT powder (10 g/day) for 5 months, prepared using EneQuick® (10 g) (The Nisshin OilliO Group, Ltd., Tokyo, Japan) mixed with 10 mL of milk and 20 g of jam (AOHATA CROPORATION, Ltd., Hiroshima, Japan) to make an ice cream-style mousse (135 kcal, 0.4 g protein), or with 20 mL Calpis to make a Calpis mousse (136 kcal, 0.4 g protein). The nutritional contents of the MCT snacks are shown in Table 1. The daily dose of medium-chain triglycerides (MCT) was set at 10 g/day. Previous studies have shown that approximately 6 g/day of MCT is sufficient to elicit physiological and metabolic effects in older adults [16]. In the present study, we selected a slightly higher dose because our aim was also to provide an energy increase of approximately 100 kcal/day to support overall nutritional intake in residents of long-term care health facilities. We chose a dose that would not interfere with the participants’ usual food intake, ensuring that the additional energy could be consumed without reducing their habitual meal consumption.

**Table 1:** Nutritional value of snacks made with MCT powder Values are presented per serving. The table includes energy (kcal), protein (g), fat (g), carbohydrate (g), salt equivalent (g), and medium-chain triglyceride oil (g). The MCT snacks were prepared using EneQuick® powder (Nisshin OilliO Group, Ltd., Tokyo, Japan). The ice-cream-style mousse was made with milk and jam, and the Calpis mousse was made with Calpis. EneQuick® powder was incorporated into the formulation of each snack type. t-values and 95% confidence intervals are shown in Supplementary Table 1. MCT: Medium-chain triglycerides

Ingredients	Ice-cream Style Mousse	Calpis Mousse	EneQuick®
Energy (kcal)	129	136	90
Protein (g)	0.4	0.4	0
Fats (g)	10.4	10.0	10.0
Carbohydrate (g)	8.5	11.0	0.0
Salt equivalent (g)	0	0.03	0
Medium-chain triglyceride oil (g)	-	-	9.0

The snacks were provided daily at 3 p.m. The control group received only the standard meals offered at the facility, identical to the meals given to the intervention group. Nutritional intake values for both groups are provided in Table 2. Both groups received energy and protein based on the estimated requirements calculated using the 2020 edition of the Dietary Reference Intake for the Japanese, which were recalculated monthly [17].

**Table 2:** Amount of nutrition provided to intervention and control groups The table presents the average daily intake of energy (kcal), protein (g), fat (g), carbohydrates (g), and other nutrients provided to the intervention and control groups during the study period. Values are expressed as mean ± SD. MCT supplementation was included only in the intervention group.

Ingredients	MCT Oil	Control	p-value	t-value	95% Confidence Intervals (Lower-Upper)
Energy (kcal)	1368.6±111.7	1396.3±123.0	0.362	−0.9478	−143.22 - 56.35
Protein (g)	50.5±2.0	51.3±1.9	0.188	−1.3867	−2.667 - 0.575
Fat (g)	43.5±0.2	43.5±0.3	0.928	0.0928	−0.231 - 0.252
Carbohydrates (g)	199.9±27.5	196.6±34.5	0.829	0.2200	−25.85 - 31.752
Salt Equivalent (g)	7.7±0.3	7.7±0.3	-	-	-

Both groups received energy and protein based on the estimated requirements calculated using the 2020 edition of the Dietary Reference Intake for the Japanese[17], which were recalculated monthly.

Meal Intake Assessment

In usual clinical settings, participants’ dietary intake (breakfast, lunch, and dinner) was assessed by visual estimation for each tray by both nurses and dietitians. The dietitians estimated the intake based on the proportion of the total meal consumed. Data is recorded as a percentage (0%-100%), and daily averages were used. Intake of 0% indicated no intake, and 100% indicated full consumption.

Test Snack Intake Assessment

Test snack consumption was evaluated by the dietitian after each snack session, based on the amount left on the tray.

Other variables

From the medical records, data on age, sex, height, care level, and comorbidities were collected. Ambulatory status was categorized into three levels: “independent” for those able to walk without assistance, “aid required” for those needing a cane or walker, or “wheelchair” for those always using a wheelchair.

Anthropometric Measurements

Body weight was measured monthly by nursing staff. Height was obtained from facility records, and BMI was calculated as weight (kg) divided by the square of height (m²).

$BMI = \frac{weight~(kg)}{[height~(m)]^{2}}$

Calf circumference (CC), arm circumference (AC), and triceps skinfold thickness (TSF) were measured using a non-stretchable measuring tape (Insa Tape®, Abbott Japan LLC, Tokyo, Japan) and an Adipometer® (Abbott Japan LLC, Tokyo, Japan), in accordance with the Japanese Anthropometric Reference Data (JARD 2001)[18].

CC was measured once at the widest part of the calf with the tape positioned horizontally without applying excessive pressure. AC was measured once on the non-dominant arm at the midpoint between the acromion and the olecranon, ensuring minimal compression of the soft tissue. TSF was measured once at the same midpoint on the posterior aspect of the non-dominant upper arm, using the Adipometer®, with the reading taken approximately two seconds after applying the caliper.

$AMC~(cm) = AC~(cm) - \pi \times \frac{TSF~(mm)}{10}$

All parameters were taken by researchers who were qualified dieticians and had the skills necessary for accurate measurements. These methods were selected because they provide simple, non-invasive, and cost-effective assessments that are especially useful in settings where body weight cannot be measured, such as among residents who are bedridden. To minimize participant burden, all measurements were performed only once.

Grip Strength

Grip strength was measured during physical performance testing by a physiotherapist using a digital handgrip dynamometer (N-Force HG-200, CORVETTE CROPORATION, Ltd., Wakayama, Japan, Smedley type). The dominant hand was used unless paralyzed. Participants were seated, with their shoulders in a neutral position, elbows flexed at 90°, and forearms in a neutral position.

Food Intake LEVEL Scale (FILS)

Swallowing ability was assessed using a 10-level FILS: levels 1-3 indicated no oral intake, levels 4-6 indicated partial oral intake with alternative nutrition, levels 7-9 indicated full oral intake, and level 10 indicated no swallowing problem. Dietitians assessed and recorded the levels[19]

Nutritional Status

Nutritional status was assessed using MNA®-SF, with scores interpreted as follows: 0-7: malnourished, 8-11: at risk of malnutrition, and 12-14: normal nutrition.

Cognitive Function

Cognitive function was assessed using the Hasegawa Dementia Scale-Revised (HDS-R), conducted every 3 months by a physiotherapist[20]. A score ≤20 (out of 30) indicated suspected dementia.

Ethical considerations

All participants provided written and oral informed consent. The Ethics Committee of the Prefectural University of Hiroshima approved the study protocol (Approval No. 23HH003).

Statistical analysis

Baseline comparisons between the intervention and control groups were performed using the Mann-Whitney U test and chi-squared test. Within-group pre-post comparisons were analyzed using the Wilcoxon signed-rank tests. Based on a previous study, analysis of covariance (ANCOVA) was performed with baseline adjustment for anthropometric data, MNA®-SF score, grip strength, and HDS-R score. The mean differences and rates of change before and after intervention were calculated. Data are expressed as mean ± standard deviation (%). Statistical analysis was performed using EZR version 4.40, with significance set at p < 0.05.

## Results

Participants’ basic characteristics

**Figure 1:**
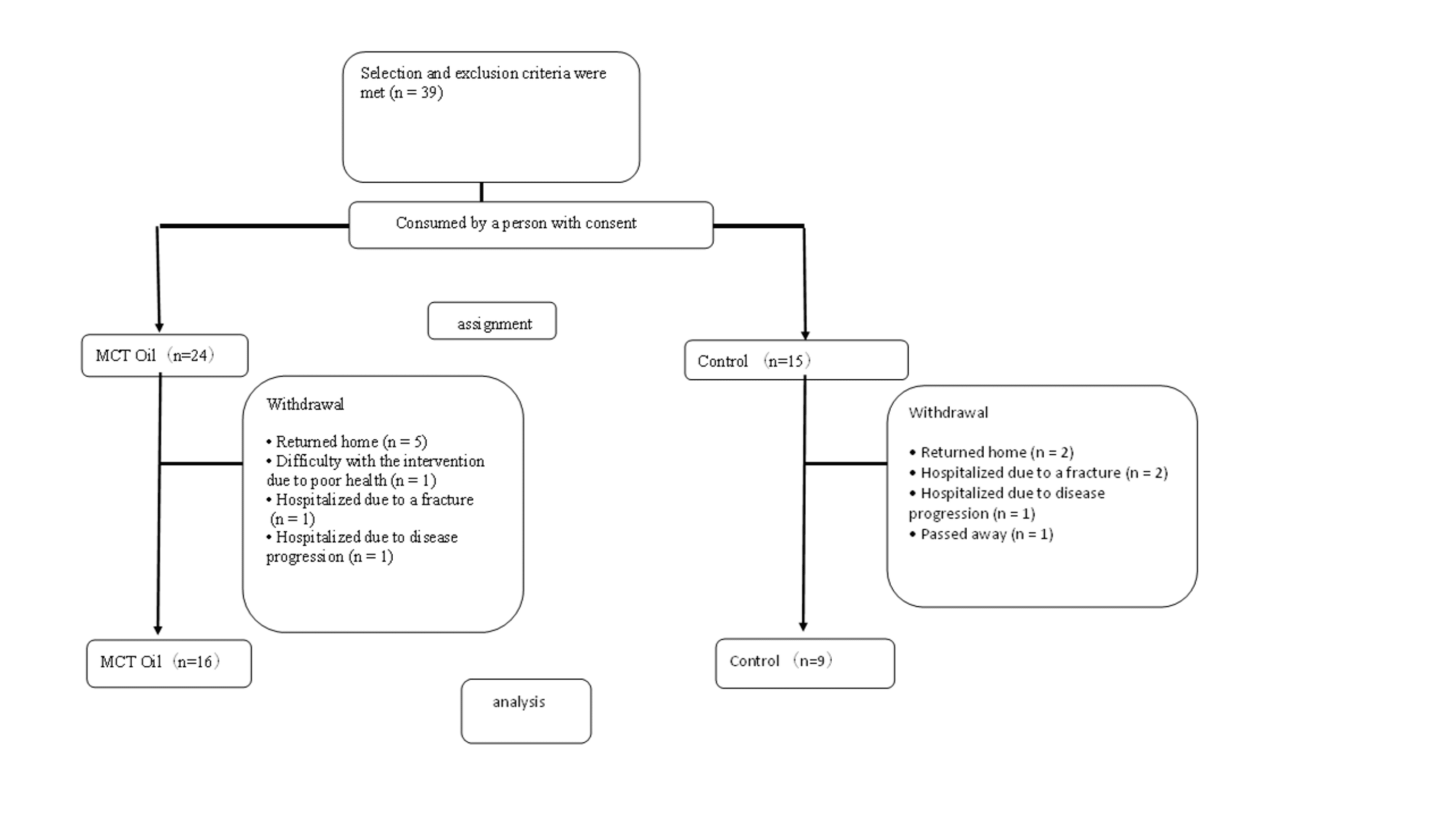
The participant flow, including enrollment, allocation, follow-up, and final analysis.

We enrolled 39 in the trial (Figure 1), after accounting for dropouts, data from sixteen participants in the intervention group and nine participants in the control group were analyzed. Table 3 shows the physical characteristics, including age, sex, care level, and medical history, of all participants and those who remained after dropout.

**Table 3:** Physical characteristics of the participants Summary of physical characteristics of participants in the study. Values are expressed as mean ± SD or number (%). MCT: medium-chain triglyceride

Characteristic	Item	MCT Oil (n=16)	Control (n=9)	p-value	χ^2^ value	95% Confidence Intervals (Lower-Upper)	
Age	(years)	88.1±4.9	88.1±6.0	0.985	-	−5.316-5.219	
Sex	Man	1 ( 6.2)	3 (33.3)	0.116	3.144	0.449 - 417.94	
	Women	15 (93.8)	6 (66.7)	-	-	-	
Care level	Not applicable	2.8±1.1	3.2±1.5	0.505	-	−1.698 - 0.879	
Disease (Multiple answer allowed)	Hypertension	4 (25.0)	1 (11.1)	0.6206	0.694	0.007 - 4.969	
	Orthopedic	9 (37.5)	4 (44.4)	0.688	0.322	0.087 - 4.274	
	Cerebrovascular	3 (12.5)	2 (22.2)	0.688	0.043	0.084 - 13.65	
	Dementia	5 (20.8)	3 (33.3)	0.915	0.011	0.125 - 8.32	
	Cardiovascular disease	3 (12.5)	6 (66.7)	0.031	5.740	0.997 - 83.45	
	Mental disorder	1 (4.2)	1 (11.1)	1.000	0.185	0.021 - 156.6	
	Malignant tumor	0 (0.0)	0 (0.0)	-			
	Others	2 (8.3)	1 (11.1)	1.000	0.010	0.013 - 19.52	
Walking condition	Walking unassisted	3 (18.7)	1 (11.1)	1.000	0.250	0.009 - 8.39	
	Use of aids	4 (25.0)	3 (33.3)	0.673	0.198	0.013 - 19.52	
	Wheelchair	9 (56.3)	5 (55.6)	1.000	0.001	0.142 - 6.97	

Table 4 compares baseline physical measurements, nutritional status, physical function, and cognitive function between the intervention and control groups.

**Table 4:** Comparison of body measurements and nutritional status, and physical and cognitive abilities before the intervention Comparison of body measurements, nutritional status, and physical and cognitive abilities before the intervention. BMI: body mass index; CC: calf circumference; AC: arm circumference; TSF: triceps skinfold thickness; AMC: arm muscle circumference; FILS: food intake level scale; MNA-SF: Mini Nutritional Assessment–Short Form; HDS-R: Hasegawa’s Dementia Scale–Revised

Characteristic	Measure	MCT Oil (n=16)	Control (n=9)	p-value	t-value	95% Confidence Intervals (Lower-Upper)
BMI	(kg/m^2^)	18.8±2.2	20.5±2.8	0.122	−1.648	−4.134 - 0.545
CC	(cm)	29.2±2.4	31.1±3.3	0.163	−1.481	−4.813 - 0.903
AC	(cm)	22.0±1.8	23.1±2.3	0.270	−1.153	−3.127 - 0.949
TSF	(mm)	16.4±5.7	16.4±6.0	0.979	−0.003	−5.549 - 5.536
AMC	(cm)	16.9±1.9	17.9±1.7	0.189	−1.368	−2.711 - 0.576
FILS	(point)	9.0±1.1	8.9±1.4	0.845	0.200	−1.089 - 1.311
MNA-SF	(point)	7.4±1.6	7.2±2.1	0.859	0.181	−1.675 - 1.981
Energy requirement	(kcal)	1349.9±213.1	1384.5±229.5	0.728	−0.354	−243.2 - 173.9
Protein requirement	(g)	48.5±6.9	51.1±6.7	0.393	−0.876	−8.854 - 3.663
Dietary intake	(%)	87.5±20	96.7±7.0	0.115	−1.647	−20.759 - 2.426
Test snack intake	(%)	98.70	-	-	-	-
Grip strength	(kg)	9.9±3.9	11.6±6.2	0.478	−0.734	−6.690 - 3.329
HDS-R	(point)	14.7±7.0	18.8±4.8	0.301	−1.107	−12.62 - 4.456

No significant differences were observed between the groups in age, sex, or care level, physical measurements, food form, physical function, or cognitive function at baseline.

Comparison of anthropometric and nutritional status before and after intervention

Table 5 shows the within-group comparisons before and after intervention in both the intervention and control groups.

**Table 5:** Pre- and post-intervention within-group comparisons of endpoints in the intake and control groups Comparison of baselines and post-intervention values of outcome variables within the intake and control groups. Values are expressed as mean ± SD, statistical significance assessed using paired t-test. BMI: body mass index; CC: calf circumference; AC: arm circumference; TSF: triceps skinfold thickness; AMC: arm muscle circumference; FILS: food intake level scale; MNA-SF: Mini Nutritional Assessment–Short Form; HDS-R: Hasegawa’s Dementia Scale–Revised

Characteristic	Measure	MCT Oil (n=16)						Control (n=9)					
		Before	3 month	5 month	p-value	t-value	95% confidence intervals (Lower-Upper)	Before	3 month	5 month	p-value	t-value	95% confidence intervals (Lower-Upper)
BMI	(kg/m^2^)	18.8±2.2	19.0±1.8	19.7±2.3	0.002	-3.629	-1.468 - -0.381	20.5±2.8	20.2±2.4	20.2±2.7	0.476	-0.767	-0.780 - 1.559
CC	(cm)	29.2±2.4	29.0±3.0	29.6±2.2	0.442	0.789	-0.691 - 1.503	31.1±3.3	29.9±2.8	30.7±2.1	0.491	-0.722	-1.631 - 0.853
AC	(cm)	22.0±1.8	22.1±2.0	22.0±2.0	1.000	0.000	-0.766 - 0.766	23.1±2.3	22.4±2.2	22.2±2.3	0.074	-2.053	0.853 - 0.114
TSF	(mm)	15.8±5.7	13.8±6.1	13.3±6.4	0.011	-2.882	-5.327 - -0.798	15.3±6.4	14.9±6.3	13.3±4.4	0.172	-1.500	-5.075 - -0.798
AMC	(cm)	16.9±1.9	17.8±0.9	17.8±1.8	0.128	1.610	-0.312 - 2.235	18.3±1.2	17.7±0.8	18.0±1.4	0.576	-0.583	-1.459 - 0.870
FILS	（point）	9.0±1.1	9.1±1.1	9.0±1.1	1.000	0.000	-0.389 - 0.389	8.9±1.4	8.9±1.4	8.9±1.4	1.000	0.000	-0.389 - 0.389
MNA-SF	（point）	7.4±1.6	9.0±1.2	9.7±1.4	<0.001	7.738	1.675 - 2.949	7.2±2.1	8.9±1.5	9.6±1.6	0.002	4.221	1.059 - 3.608
Grip strength	(kg)	9.9±3.9	10.6±5.4	11.1±5.2	0.049	2.192	0.033 - 2.342	11.6±6.2	11.1±6.5	10.4±6.5	0.159	-1.552	-2.762 - 0.539
HDS-R	(point)	14.7±7.0	17.1±7.1	17.5±9.0	0.173	1.498	-1.498 - 7.053	18.8±4.8	18.8±4.8	18.0±5.4	0.749	-0.351	-7.548 - 6.048

In the intervention group, BMI increased from 18.8 ± 2.2 kg/m² at baseline to 19.7 ± 2.3 kg/m² after 5 months (t=-3.629, p=0.002, 95%CI (-1.468 - -0.381)); BMI increased in 14/16 participants (87.5%). In the control group, BMI decreased slightly from 20.5 ± 2.8 kg/m² at baseline to 20.2 ± 2.7 kg/m² at 5 months(t=-0.767, p=0.476, 95%CI (-0.780 - 1.559)) The MNA-SF score increased in the intervention group from 7.4 ± 1.6 to 9.7 ± 1.4 (t=7.738, p  < 0.001, 95% CI (1.675 - 2.949)), and in the control group from 7.2 ± 2.1 to 9.6 ± 1.6 (t=4.221, p=0.002, 95% CI (1.059 - 3.608)), indicating an improvement in nutritional status in both groups. No changes were observed in CC and AC in either group. TSF decreased significantly in the intervention group after 5 months (t=-2.882, p=0.011, 95%CI (-5.327 - -0.798)), whereas it did not change in the control group (t=-1.500, p=0.172, 95%CI (-5.075 - -0.798)). AMC increased in the intervention group after 5 months (t=1.609, p=0.128, 95% CI (-0.312 - 2.235)), whereas no significant change was observed in the control group (t=-0.583, p=0.576, 95%CI (-1.459 - 0.870)).

Comparison of physical and cognitive function before and after intervention

Grip strength in the intervention group increased from 9.9 ± 3.9 kg at baseline to 11.1 ± 5.2 kg after 5 months (t=2.192, p=0.049, 95% CI (0.033 - 2.342)). In the control group, grip strength changed insignificantly from 11.6 ± 6.2 kg to 10.4 ± 6.5 kg (t=-1.552, p=0.159, 95% CI (-2.762 - 0.539)). HDS-R scores increased from 14.7 ± 7.0 to 17.5 ± 9.0 in the intervention group (t=1.498, p=0.173, 95% CI (-1.498-7.053)) and decreased from 18.8 ± 4.8 to 18.0 ± 5.4 in the control group (t=-0.351, p=0.749, 95% CI (-7.548-6.048)); however, no significant changes were observed in either group.

Changes from baseline to 5 months

Table 6 provides the changes in anthropometric values from baseline to 5 months and the differences between groups.

**Table 6:** Changes between pre-intervention and 5 months post-intervention in each group Mean differences in selected parameters between baseline and 5-month follow-up. Values are expressed as mean SD±; a paired t-test was used to assess significance. Differences adjusted for baseline showed 25,75 percentile values. BMI: body mass index; CC: calf circumference; AC: arm circumference; TSF: triceps skinfold thickness; AMC: arm muscle circumference; MNA-SF: Mini Nutritional Assessment–Short Form; HDS-R: Hasegawa’s Dementia Scale–Revised

Characteristic	Measure	Group	n	Baseline	5 months	Change	t-value	95% Confidence Intervals (Lower-Upper)	Baseline-Adjusted Change (25, 75th Percentiles) Coefficient (95% CI)	F-Value	Percent Change(％)	t-value	95% Confidence Intervals (Lower-Upper)
BMI	(kg/m^2^)	MCT Oil	16	18.8±2.2	19.7±2.3	0.9±1.0	Not applicable	Not applicable	(0.8, 1.2)	Not applicable	5.1	Not applicable	Not applicable
		Control	9	20.5±2.8	20.2±2.7	−0.4±1.5	Not applicable	Not applicable	(−0.7, 1.4)	Not applicable	−1.7	Not applicable	Not applicable
		p-value	Not applicable			0.039	2.315	0.079 - 2.549	0.050 (−0.292 - 0.000)	3.928	0.022	2.582	0.011- 0.124
CC	(cm)	MCT Oil	16	29.2±2.4	29.6±2.2	0.4±2.1	Not applicable	Not applicable	(−1.1, 1.1)	Not applicable	1.7	Not applicable	Not applicable
		Control	9	31.1±3.3	30.7±2.1	−0.4±1.6	Not applicable	Not applicable	(−1.0, 0.0)	Not applicable	−0.8	Not applicable	Not applicable
		p-value	Not applicable			0.298	1.067	−0.758 - 2.348	0.999 (−1.414 - 1.416)	7.325	0.363	0.930	−0.030 - 0.079
AC	(cm)	MCT Oil	16	22.0±1.8	22.0±2.0	0±1.5	Not applicable	Not applicable	(−1.0, 0.6)	Not applicable	0.2	Not applicable	Not applicable
		Control	9	23.1±2.3	22.2±2.3	-0.9±1.3	Not applicable	Not applicable	(−1.5, 0)	Not applicable	−3.8	Not applicable	Not applicable
		p-value	Not applicable			0.127	1.603	−0.289 - 2.132	0.234 (−1.972 - 0.509)	2.067	0.137	1.549	−0.013 - 0.093
TSF	(mm)	MCT Oil	16	16.4±6.2	13.3±6.4	-3.1±4.3	Not applicable	Not applicable	(−6.0, 1.5)	Not applicable	−18.5	Not applicable	Not applicable
		Control	9	15.3±6.4	13.3±4.4	−2.0±4.0	Not applicable	Not applicable	(−2, 0)	Not applicable	−9.4	Not applicable	Not applicable
		p-value	Not applicable			0.541	−0.623	−4.650 - 2.525	0.634 (−2.578 - 4.143)	2.493	0.373	−0.911	−0.297 - 0.116
AMC	(cm)	MCT Oil	16	16.9±1.9	17.8±1.8	1.0±2.4	Not applicable	Not applicable	(−0.0, 1.6)	Not applicable	7.2	Not applicable	Not applicable
		Control	9	18.3±1.2	18.0±1.4	-0.3±1.5	Not applicable	Not applicable	(−1.3, 0)	Not applicable	−1.4	Not applicable	Not applicable
		p-value	Not applicable			0.122	1.605	−0.364 - 2.876	0.912 (−1.258 - 1.400)	16.98	0.097	1.728	−0.017 - 0.189
MNA-SF	(point)	MCT Oil	16	7.4±1.6	9.7±1.4	2.3±1.2	Not applicable	Not applicable	(−0.0, 0)	Not applicable	31.4	Not applicable	Not applicable
		Control	9	7.2±2.1	9.6±1.6	2.3±1.7	Not applicable	Not applicable	(−0.3, 3.0)	Not applicable	32.3	Not applicable	Not applicable
		p-value	Not applicable			0.974	-0.033	−1.380 - 1.339	0.928 (−1.041 - 0.953)	5.336	0.72	−0.366	−0.270 - 0.192
Strength grip	(kg)	MCT Oil	16	9.9±3.9	11.1±5.2	1.2±2.2	Not applicable	Not applicable	(−0.3, 3.0)	Not applicable	11.7	Not applicable	Not applicable
		Control	9	11.6±6.2	10.4±6.5	-1.1±2.1	Not applicable	Not applicable	(−1.0, 0.0)	Not applicable	−10.5	Not applicable	Not applicable
		p-value	Not applicable			0.020	2.561	0.0403- 4.194	0.027 (−2.282 - −0.154)	3.962	0.069	1.9605	−0.019- 0.463
HDS-R	(point)	MCT Oil	9	14.7±7.0	17.5±9.0	2.8±5.6	Not applicable	Not applicable	(3.0, 7.0)	Not applicable	21.0	Not applicable	Not applicable
		Control	4	18.8±4.8	18.0±5.4	-0.8±4.2	Not applicable	Not applicable	(−4.0, 1.7)	Not applicable	−2.7	Not applicable	Not applicable
		p-value	Not applicable			0.249	1.105	−0.346 - 0.995	0.323 (−11.26 - 4.098)	0.5715	0.364	1.2472	−3.054 - 10.10

An ANCOVA adjusted for baseline values showed that the change in BMI was 0.9 ± 1.0 kg/m² in the intervention group and −0.4 ± 1.5 kg/m² in the control group (F-value=3.928, p=0.050, 95% CI (−0.292 - 0.000)), indicating a trend toward increased BMI in the intervention group. Grip strength increased by 1.2 ± 2.2 kg in the intervention group and decreased by −1.1 ± 2.1 kg in the control group (F-value=3.962, p=0.027, 95% CI (−2.282 - −0.154)), demonstrating a significant difference after adjustment, with improved grip strength in the intervention group. No significant differences were observed in other parameters, though MNA-SF scores increased in both groups: (intervention:2.3 ± 1.2 vs. control: 2.3 ± 1.7)(F-value=5.336, p=0.928, 95% CI (−1.041 - 0.953)). With regard to cognitive function, the change in HDS-R scores was 2.8 ± 5.6 in the intervention group and −0.8 ± 4.2 in the control group (F-value = 0.5715, p = 0.323, 95% CI (−11.26 - 4.098)), indicating no significant between-group differences.

BMI increased by 5.1% in the intervention group, whereas it decreased by 1.7% in the control group, demonstrating a significant difference between the groups (t = 2.582, p = 0.022, 95% CI (0.011-0.124)). Grip strength increased by 11.7% in the intervention group and decreased by 10.5% in the control group, showing a trend toward significance (t = 1.961, p = 0.069, 95% CI (−0.019-0.463)). The MNA-SF score increased by 31.4% in the intervention group and by 32.4% in the control group (t = −0.367, p = 0.720, 95% CI (−0.270-0.192)). In contrast, the HDS-R score increased by 21.0% in the intervention group but decreased by 2.7% in the control group (t = 1.247, p = 0.364, 95% CI (−3.054-10.10)).

## Discussion

When a snack containing 10 g of MCT powder per day was provided for 5 months in addition to regular meals, improvements in BMI and grip strength were observed in undernourished older adults residing in long-term care facilities. AMC values tended to increase, whereas TSF, an indicator of fat mass, decreased. In contrast, the control group, which did not receive MCT-supplemented snacks, showed a slight decrease in BMI after 5 months and no change in grip strength. These findings suggest that the favorable outcomes in the intervention group may be attributed to the addition of dietary MCT. However, MNA-SF scores improved in both the intervention and control groups. Because the degree of improvement was similar between groups, it was difficult to determine whether the enhancement in nutritional status was specifically attributable to MCT supplementation or to other factors such as regular nutritional care provided within the facility. Although no statistically significant differences were observed in cognitive function, scores increased in the intervention group, whereas they declined in the control group. The lack of significant changes in cognitive function may be related to the relatively short intervention duration and the reduced sample size due to participant withdrawal, which may have limited the statistical power to detect subtle effects. Several reports have shown that MCT intake, compared to Long-chain triglycerides (LCT), increases postprandial energy expenditure [14].Grip strength increased significantly by 11.7% after covariate-adjusted analysis using baseline values. Previous studies have suggested that MCTs enhance ghrelin secretion, a hormone that strongly stimulates growth hormone release and promotes skeletal muscle protein synthesis via Insulin-like Growth Factors (IGF-1), as well as contributing to weight gain through increased appetite [20]. Additionally, supplementation with MCTs (C8:0 and C10:0) has been reported to improve ADL, cognitive, and feeding function in patients with sarcopenia, even without changes in BMI, when compared with LCT [21]. In our study, increased nutritional intake led to changes in BMI. The reported benefits of MCTs, particularly medium-chain fatty acids (MCFA) (C8:0) and acyl-ghrelin, suggest that the observed effects may be attributed to these substances [22]. Although the MCT powder used in this study did not specify its MCFA content, most commercially available MCTs consist primarily of C8:0 and C10:0 [23].Similarly, the product used in this study consisted of approximately 90% medium-chain fatty acids (Table 1). Despite the differences in the form of intake (supplement vs. part of a meal), the effects are likely to be consistent. The improved grip strength observed in this study aligns with previous findings by Kojima et al., who reported increased grip strength in community-dwelling older adults receiving 6 g/day of MCTs [16]. Under various pathological conditions, MCT supplementation has been shown to enhance muscle mass and function through mitochondrial biogenesis, increased protein synthesis, and decreased protein degradation [24]. Dietary MCTs are hydrolyzed to MCFAs by lingual and gastric lipases. Acyl ghrelin, part of the appetite-stimulating hormone ghrelin, which is produced in the stomach, cannot be produced without MCFAs. Acyl ghrelin has a very important role, as it stimulates the release of growth hormone, and growth hormone increases muscle mass. Muscle mass is maintained by balancing synthesis and breakdown; however, with aging, both mass and synthesis rates decline [25]. In our study, there was a difference in the amount of protein provided relative to the protein requirements of the two groups. The control group provided 51.3 g of protein compared to the intervention group, for a protein requirement of 51.1 g. There was little increase in protein intake. Sarcopenia, characterized by reduced skeletal muscle mass, increases the risk of falls and undernutrition, and is exacerbated by insufficient protein intake [26]. Although we did not measure acyl-ghrelin concentrations and thus could not confirm the precise mechanism, the observed increase in grip strength may be due to increased energy intake or MCT supplementation. Although MCT intake may improve muscle strength and function, it may not lead to changes in muscle mass [27]. Given the advanced average age of the participants (88 years), this effect resulted from suppression of muscle protein degradation. MCTs provide approximately 8.6 kcal per gram and enhance appetite, promoting weight gain. Side effects are rare but may include abdominal pain or diarrhea when consumed in large amounts. The MCT powder used in this study had minimal taste and odor, dissolved easily in meals or beverages without altering the flavor, and was well tolerated by the participants [28]. No participants withdrew due to adverse effects or taste issues. Malnutrition in older adults has complex and overlapping causes, including social, psychological, medical, and age-related factors, and recovery is often difficult [29]. The GLIM criteria include three stages-identification of nutritional risk, diagnosis of malnutrition, and severity assessment-which are useful for identifying malnutrition causes and guiding appropriate interventions [5]. In long-term care insurance facilities, improving BMI reduces morbidity and mortality and enhances physical functions such as walking ability. Nutritional interventions using ONSs are effective for improving function [30]. In our study, MCT-containing ONSs had a positive effect on body weight without altering food intake volume, suggesting they may be effective in improving the nutritional and functional status of undernourished older individuals.

This study had several limitations. As participation was based on patient preference, this was a non-randomized study, and the intervention group likely included more cooperative individuals with a better understanding of nutritional therapy, introducing potential bias. Additionally, both groups experienced participant dropouts, resulting in small final sample sizes that limit the statistical power and generalizability of the findings. Therefore, the present results should be interpreted with caution. To more conclusively determine the effectiveness of MCT supplementation for residents of long-term care facilities, large-scale multicenter randomized controlled trials are warranted.

## Conclusions

This study demonstrated that providing one snack containing MCTs (10 g per day) for 5 months, in addition to regular meals, led to improvements in BMI and grip strength in undernourished older adults residing in long-term care facilities. Although MNA-SF scores improved in both the intervention and control groups, the similar degree of improvement made it difficult to determine the specific contribution of MCT supplementation to this outcome. No significant changes were observed in cognitive function, which may be attributed to the relatively short intervention period and the reduced sample size resulting from participant withdrawal. Nevertheless, the improvements observed in certain aspects of physical function and nutritional status suggest that snacks containing MCT powder may support nutritional management in older adults, particularly by increasing energy intake and helping preserve muscle function. These effects are likely related to the physiological properties of MCTs, such as promoting energy utilization and suppressing muscle protein breakdown, and may be realized when overall food intake is maintained. Further large-scale studies are warranted to clarify the effects of MCT supplementation on cognitive function and broader nutritional outcomes.

## Appendices

**Table 7:** t-values and 95% confidence intervals and F-values for changes between pre-intervention and 5 months post-intervention t-values and 95% confidence intervals and F-values are shown in Supplementary Table 6.

Characteristic	Measure		t-value	95% Confidence Intervals (Lower-Upper)
	BMI = \dfrac{\text{weight (kg)}}{[\text{height (m)}]^2}	Change	2.315	0.079 - 2.549
		Percentage	2.582	0.011- 0.124
		F-value	3.928	
Body Measurement	CC(cm)	Change	1.067	−0.758 - 2.348
		Percentage	0.930	−0.030 - 0.079
		F-value	7.325	
	AC(cm)	Change	1.603	−0.289 - 2.132
		Percentage	1.549	−0.013 - 0.093
		F-value	2.067	
	TSF(mm)	Change	−0.623	−4.650 - 2.525
		Percentage	−0.911	−0.297 - 0.116
		F-value	2.493	
	AMC = AC - \pi \times \dfrac{TSF}{10}	Change	1.605	−0.364 - 2.876
		Percentage	1.728	−0.017 - 0.189
		F-value	16.980	
Nutritional Status	MNA-SF (point)	Change	−0.033	−1.380 - 1.339
		Percentage	−0.366	−0.270 - 0.192
		F-value	5.336	
Physical function	Grip strength (kg)	Change	2.561	0.0403- 4.194
		Percentage	1.961	−0.019- 0.463
		F-value	3.962	
Cognitive function	HDS-R (point)	Change	1.105	−0.346 - 0.995
		Percentage	1.247	−3.054 - 10.10
		F-value	0.572	

**Table 8:** t-values and 95% confidence intervals for pre- and post-intervention within-group comparisons of endpoints in the intake and control groups t-values and 95% confidence intervals are shown in Supplementary Table 4.

		MCT Oil (n=16)		Control (n=9)	
Characteristic	Measure	t-value	95% confidence intervals (Lower-Upper)	t-value	95% confidence intervals (Lower-Upper)
	BMI = \dfrac{\text{weight (kg)}}{[\text{height (m)}]^2}	−3.629	−1.468 - −0.381	−0.767	−0.780 - 1.559
Body Measurement	CC (cm)	0.789	−0.691 - 1.503	−0.722	−1.631 - 0.853
	AC (cm)	0.000	−0.766 - 0.766	−2.053	0.853 - 0.114
	TSF (mm)	−2.882	−5.327 - −0.798	−1.500	−5.075 - −0.798
	AMC = AC - \pi \times \dfrac{TSF}{10}	1.610	−0.312 - 2.235	−0.583	−1.459 - 0.870
food form	FILS	0.000	−0.389 - 0.389	0.000	−0.389 - 0.389
Nutritional Status	MNA-SF(point)	7.738	1.675 - 2.949	4.221	1.059 - 3.608
Physical function	Grip strength (kg)	2.192	0.033 - 2.342	−1.552	−2.762 - 0.539
Cognitive function	HDS-R(point)	1.498	−1.498 - 7.053	−0.351	−7.548 - 6.048

**Table 9:** t-values and 95% confidence intervals for comparison of body measurements and nutritional status and physical and cognitive abilities before the intervention t-values and 95% confidence intervals are shown in Supplementary Table 3.

Characteristic	Measure	t-value	95% Confidence Intervals (Lower-Upper)
	BMI = \dfrac{\text{weight (kg)}}{[\text{height (m)}]^2}	−1.648	−4.134 - 0.545
Body Measurement	CC(cm)	−1.481	−4.813 - 0.903
	AC(cm)	−1.153	−3.127 - 0.949
	TSF(mm)	−0.003	−5.549 - 5.536
	AMC = AC - \pi \times \dfrac{TSF}{10}	−1.368	−2.711 - 0.576
Food from	FILS	0.200	−1.089 - 1.311
Nutritional status	MNA-SF (point)	0.181	−1.675 - 1.981
Energy requirement	(kcal)	−0.354	−243.2 - 173.9
Protein requirement	(g)	−0.876	−8.854 - 3.663
Dietary intake	(%)	−1.647	−20.759 - 2.426
Test Snack Intake	(%)	-	-
Physical function	Grip strength (kg)	−0.734	−6.690 - 3.329
Cognitive function	HDS-R(point)	−1.107	−12.62 - 4.456

**Table 10:** t-values and 95% confidence intervals for the amount of nutrition provided to intervention and control groups t-values and 95% confidence intervals are shown in Supplementary Table 1.

Ingredients	t-value	95% Confidence Intervals (Lower-Upper)
Energy (kcal)	−0.9478	−143.22 - 56.35
Protein (g)	−1.3867	−2.667 - 0.575
Fat (g)	0.0928	−0.231 - 0.252
Carbohydrates (g)	0.2200	−25.85 - 31.752
Salt Equivalent (g)	-	-
